# Linking volatile metabolites from bacterial pathogens to exhaled breath condensate of people with cystic fibrosis

**DOI:** 10.1099/mic.0.001536

**Published:** 2025-02-20

**Authors:** P. Hansani Karunarathne, Christopher Bridges, Lacy Remisoski, Madisen Crane, Claudia Soria Casanova, Samantha N. Kinne, Alicia L. Castillo Bahena, Marissa Gil, Lienwil Padillo, Gabriel Querido, Jenna Mielke, Marc McClelland, Doug Conrad, Robert A. Quinn

**Affiliations:** 1 Department of Biochemistry and Molecular Biology, Michigan State University, East Lansing, MI, USA; 2 Corewell Health, Grand Rapids, MI, USA; 3 Department of Medicine, University of California San Diego, La Jolla, CA, USA; 4 Microbiology, Genetics and Immunology, Michigan State University, East Lansing, MI, USA

**Keywords:** cystic fibrosis, pathogens, cough breath, exhaled breath condensate, volatile organic compounds

## Abstract

Obtaining sputum samples from people with cystic fibrosis (pwCF) for microbiology has become challenging due to the positive clinical effects of the cystic fibrosis transmembrane conductance regulator modulator therapy, elexacaftor-tezacaftor-ivacaftor (ETI). Although ETI improves lung function and reduces sputum production, recent data shows that bacterial pathogens persist, making continued monitoring of infection important. As an alternative to sputum sampling, this study developed a non-invasive technique called ‘Cough Breath’ (CB) to identify volatile organic compounds (VOCs) in exhaled breath condensate (EBC) and link them to cystic fibrosis (CF) bacterial pathogens using purge and trap GC-MS. The CB culturing approach was able to isolate pathogens from expectorated particulates simultaneously with EBC collection; however, culturing positivity was low, with 6% of samples collected (*n*=47) positive for either *Pseudomonas aeruginosa* or *Staphylococcus aureus*. From EBC, we identified VOCs matching those uniquely produced by *P. aeruginosa* (7), *S. aureus* (12), *Achromobacter xylosoxidans* (8) and *Granulicatella adiacens* (2); however, the overall detection rate was also low. Expanding to VOCs produced across multiple pathogens identified 30 frequently detected in the EBC of pwCF, including 2,3-pentanedione, propyl pyruvate, oxalic acid diallyl ester, methyl isobutyl ketone, methyl nitrate, 2-propenal, acetonitrile, acetoin and 2,3-butanedione. Comparing isolate volatilomes and EBC samples from the same pwCF enhanced detection rates with key VOCs, such as 2,3-pentanedione (86%) and propyl pyruvate (83%), in * P. aeruginosa* isolates. Further investigation showed that VOC production differed across strains and at different growth phases, creating variability that may explain the overall low EBC detection rate. Although this study successfully cultured CF pathogens from cough particulates and matched their unique VOCs in EBC samples, our results indicate that microbial volatiles more generally indicative of infection, such as 2,3-pentanedione, may have the most utility in aiding diagnostics in pwCF on ETI who have reduced sputum production in the clinic.

## Introduction

Bacterial infections and inflammation are among the most common complications that occur in cystic fibrosis (CF) and contribute to progressive lung function decline accentuated by acute pulmonary exacerbations [1,2]. Chronic airway infections from species such as *Pseudomonas aeruginosa* (PA), *Staphylococcus aureus* (SA), *Haemophilus influenzae*, members of the *Burkholderia cepacia* complex and a few others are a significant challenge for people with CF (pwCF) that negatively impact their quality of life. These well-studied bacterial pathogens are known to aggravate disease pathogenesis and are major targets of antimicrobial therapy [3,4]. However, recent research has shown that CF airway infections are polymicrobial [5,6], with a complex consortium of pathogens and other microbial residents of the upper airways infecting the lungs. This makes pathogen detection an important first step to developing appropriate clinical antimicrobial treatment courses. Their detection is traditionally performed by culturing and identifying bacterial species obtained from various CF respiratory secretions [2,7], with spontaneously expectorated sputum being the primary specimen used for clinical culturing [8,11].

However, obtaining sputum samples from pwCF has become challenging due to the positive clinical effects of a recently approved highly effective cystic fibrosis transmembrane conductance regulator (CFTR) modulator therapy, elexacaftor-tezacaftor-ivacaftor (ETI). Many pwCF taking ETI have significantly improved lung function, sweat chloride, pulmonary exacerbations and other classic symptoms [12,14]. One of the earliest consequences of ETI therapy, observed during clinical trials, was a reduction in sputum production soon after administration [15,17]. Though some subjects can still produce sputum, many have seen a significant decrease in their ability to expectorate, especially spontaneously in the clinical environment [15]. This spontaneous expectoration was a necessary aspect of routine clinical bacterial culturing but is now more challenging because of the positive effects of ETI. Whilst the beneficial effects of ETI have been life-changing for many people with this disease, it is somewhat paradoxical how it has made traditional clinical care difficult from a microbiological perspective. Furthermore, recent data shows that many subjects taking ETI are still infected with pathogens, although at a significantly reduced load, despite the improvements to lung function and symptoms [15,18, 19]. These persistent infections may continue to cause progressive airway disease, emphasizing the need for continual vigilance and rigorous monitoring of infection. Because sputum production has become much more difficult in the clinical environment, alternative methods need to be explored to detect the presence of pathogens in CF airways. Throat swabs are the primary alternative to sputum cultures and can also culture bacteria from pwCF, but these samples poorly represent the lower airways, are less sensitive than direct sputum cultures and are collected using an invasive procedure [20,22].

Breath analysis has gained more interest in the scientific community as a non-invasive alternative technique that can be used to detect airway pathogens, including in pwCF [23,28]. Some breath biomarkers, such as volatile organic compounds (VOCs), could be used to detect CF respiratory infections. VOC-based diagnostics offer significant potential as the next generation of screening tools for identifying pathogens and managing infectious diseases [29,31]. The distinct metabolic activities of different pathogenic species result in characteristic volatilomes, making them valuable indicators for diagnostic purposes [32,33]. MS-based detection of volatiles in exhaled breath has been developed for the detection of a wide variety of clinical conditions, with utility in the detection of lung cancer [34,37]. It is intuitive, then, to expand this approach to detect bacterial volatiles in those with airway infections, particularly pwCF who cannot produce sputum. A few previous studies of bacterial volatilomes have used the novel GC×GC-MS analysis approach, uncovering a diverse range of VOCs produced by different micro-organisms [38,40]. Using exhaled breath, they demonstrated the potential of microbial and breath volatile metabolites for sensitive detection and accurate identification of lung pathogens. Although exhaled breath has shown promise for the detection of pathogens in pwCF, the collection method can be cumbersome, requiring specialized equipment, limiting its translatability at the level required for traditional screening of pathogens. Therefore, alternative methods to capture the volatiles in exhaled breath are needed, especially those with convenience and utility in the clinical environment.

Exhaled breath condensate (EBC) is a collection of tiny droplets condensed from water vapour present in exhaled breath in a cold container. EBC is a promising alternative approach to VOC collection because the breath is captured by condensation, usually in a collection device that can produce approximately 1 ml of liquid. EBC contains aerosolized airway lining fluid, which carries molecules and compounds from the respiratory tract [41,42] and carries volatile compounds with the potential for detecting bacterial metabolites. Although some studies have investigated the VOCs of PA and SA clinical isolates using GC-MS analysis [38,40], there is a paucity of studies attempting to detect these microbial molecules in EBC collected from pwCF, and even fewer to that attempt to identify them as exclusively of microbial origin [43]. Additionally, a comprehensive analysis of the volatilome of the other CF pathogens, including *Stenotrophomonas maltophilia* (SM), *Achromobacter xylosoxidans* (AX), *Streptococcus* spp., *Granulicatella adiacens* (GA) and *Rothia mucilaginosa* (RM), is lacking.

Here, we characterized VOC signatures produced by CF pathogens in the EBC of pwCF, providing insights into the volatile composition associated with CF infections. Our study used a novel, dual sampling approach to detect bacterial pathogens in CF airways called ‘Cough Breath’ (CB), where a subject coughs into a microbial filter and simultaneously collects condensate from the filtered exhaled breath. This dual sampling approach takes advantage of both cough-based detection and volatilomics in a single non-invasive collection procedure.

## Methodology

### Study subjects

We recruited adult participants, aged 18 and older, who were diagnosed with CF to collect CB samples. Regulatory approval for the study was provided by the University of California San Diego (UCSD) Human Research Protection Program IRB as a parent IRB under protocol #804270, and sample collection adhered to the principles of the Declaration of Helsinki. Table 1 presents the demographic characteristics of the 69 pwCF included in the study.

**Table 1.** Demographic characteristics of pwCF

Demographic characteristic	pwCF (*n*=69)
Age (years)	34±12
BMI (kg m^−2^)	24.1±4.8
***CFTR*** ***modulators (%)***	
ETI	73
Other	10
None	17
***Gender (%)***	
Males	39
Females	61
***Infections (%)***	
PA	40
SA	44
AX	1
SM	6
*Streptococcus* spp.	4
Other#	11
None	31
***Patient status (%)***	
Stable	87
Exacerbation	13
***Pancreatic status (%)***	
Sufficient	40
Insufficient	60
pwCF on antibiotics (%)	70
Volume of EBC collected (µl)	534±66

Values are expressed as means±sd or percentages.

#Other infection types, accounting for 19% of the cohort, included *Klebsiella pneumoniae*, *Mycobacteroides abscessus*, *Burkholderia multivorans*, yeast and *Aspergillus* spp.

### EBC and CB sample collection

**Fig. 1.**
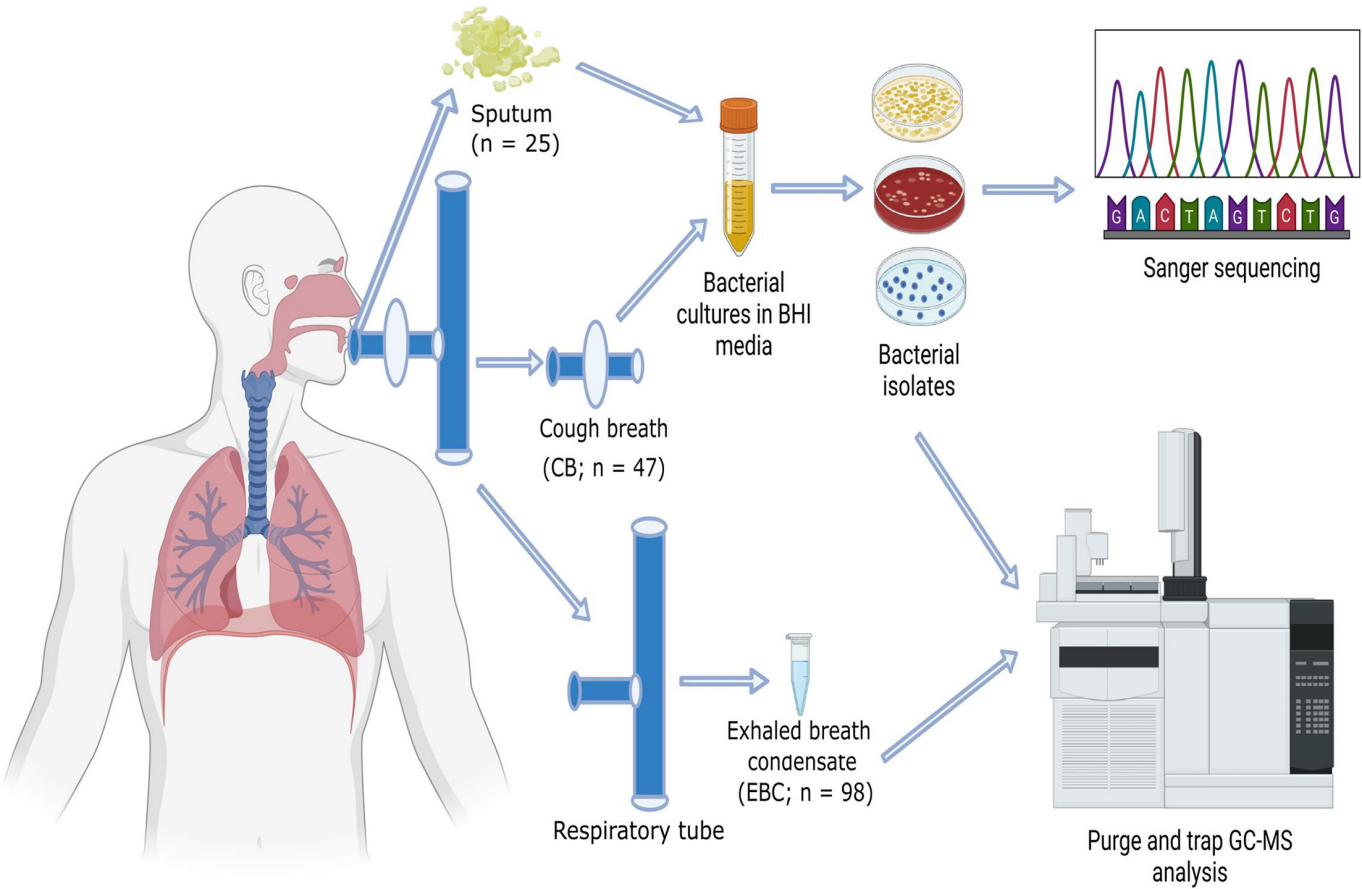
Schematic diagram demonstrating the novel CB dual sampling approach. Each subject was asked to intermittently cough into the sampling unit for a total of ten coughs whilst breathing for 5–7 min to collect EBC. If available, sputum samples (*n*=25) were also collected, and the CB filter units (*n*=47) were cultured for bacterial pathogens using rich media, then isolated on selective agar. Simultaneously, EBC samples (*n*=98) were collected and frozen for VOC analysis. The sampling approach was opportunistic, where subjects provided a CB or sputum sample, if possible, but all sampling events included an EBC collection.

Two distinct approaches for breath analysis were employed, as illustrated in Fig. 1. The sample collection was carried out with a modification to the RTube™ (Respiratory Research, Inc., Charlottesville, VA), a device designed for the collection of EBC. A bacterial filter unit was attached to the mouthpiece of the RTube in an attempt to also capture any bacteria during the CB collection. For CB dual sample collection, the subjects were asked to breathe in and out for 5–7 min through the mouthpiece with intermittent hard coughs (~10 coughs) into the bacterial filter units. The filters were carefully detached and used for culturing in non-selective bacterial media. The EBC component of the sample, which flows through the filter into the RTube and condenses, was aliquoted into 1.5 ml Eppendorf centrifuge tubes and frozen at −80 °C for further analysis with MS. The collected EBC volumes were measured by reading graduation on the sample collection tube after clearing the RTube and ranged from 500 to 800 µl across the study participants (Tables 1 and S1, available in the online Supplementary Material). The CB method was applied to 38 pwCF, with a total of 47 paired CB filter units and EBC samples collected from participants at two CF clinics: UCSD (*n*=15) and Corewell Health, Grand Rapids (*n*=32). To increase the sample size for our analysis, we included an additional 51 EBC samples that were previously collected from pwCF who did not provide CB samples. This brought the total number of EBC samples to 98, collected from 69 pwCF across the two clinics (UCSD: *n*=38; Corewell Health: *n*=60). The demographic characteristics of the study participants are provided in Table 1. To facilitate comparative analysis, sputum samples were also collected for culturing from pwCF who were still able to produce sputum.

### Isolation and identification of bacterial species in CB and sputum

To enhance the detection of VOCs associated with CF pathogens, in addition to the CB filters, we collected 25 sputum samples from pwCF to increase the likelihood of isolating target pathogens for volatilomic analysis. To culture bacteria from the CB filters, sterile brain heart infusion (BHI) broth (10 ml) was poured into the bacterial filters and shaken thoroughly using a vortex to facilitate the dissolution of bacterial cells caught in the filter units into the media. The bacteria recovered from the filters were incubated aerobically for 24 h at 37 °C with 220 r.p.m. shaking. Sputum samples were cultured and incubated under the same conditions. The resulting mixed bacterial broth cultures were inoculated onto selective agar media (i.e. *Pseudomonas* isolation agar and mannitol salt agar) and non-selective agar media (i.e. BHI, reinforced clostridial media) for isolation and purification of CF pathogens.

Identification was achieved through colony PCR amplification followed by Sanger sequencing of the 16S rRNA gene (V1-V9 gene sequence region), utilizing 27F (AGAGTTTGATCMTGGCTCAG) and 1492R (TACGGYTACCTTGTTACGACTT) primers. The PCR amplification was confirmed on 1% agarose gel electrophoresis, followed by visualizing the bands using a gel imager (Corning Axygen Gel Documentation System, USA), and the data were processed using Geneious Prime (v. 2024.0.5). Bacterial identification was carried out using the National Center for Biotechnology Information database. For the characterization of the volatilome, the following CF pathogens were isolated and identified from CB and sputum samples: PA (*n*=21; 20 from sputum, 1 from CB), SA (*n*=24; 22 from sputum, 2 from CB) and *Streptococcus* spp. (*n*=7; three from sputum, four from CB). The remaining CF pathogens were isolated exclusively from sputum, including AX (*n*=10), GA (*n*=1), SM (*n*=1) and RM (*n*=1). A detailed list of all CF isolates used in this study is provided in Table S2.

### EBC sample preparation for GC-MS analysis

The EBC samples were thawed on ice, and 500 µl of EBC was mixed with 9.5 ml of Milli-Q water to a final volume of 10 ml per sample. The samples were then transferred into 40 ml amber glass vials, each sealed with a polytetrafluoroethylene (PTFE) silicone septum screw cap (Millipore Sigma, USA) for GC-MS analysis.

### CF isolate preparation for GC-MS analysis

To investigate the volatilome of CF pathogens, bacterial isolates obtained from either sputum or CB samples, as previously described, were utilized. These CF isolates were cultured in BHI media (5 ml) and incubated aerobically at 37 °C for 24 h with 220 r.p.m. shaking. OD [OD at 600 nm (OD_600_)] was measured, and cultures were subsequently filter-sterilized using 0.22 µm filter units. Four millilitres of the filter-sterilized bacterial culture were mixed with Milli-Q water to a final volume of 10 ml per sample. The samples were then transferred into 40 ml amber glass vials, each sealed with a PTFE silicone septum screw cap (Millipore Sigma, USA) for GC-MS analysis. Additionally, 7 sterile BHI media controls, 6 Milli-Q water controls and 20 blank vials were prepared and run concurrently with the samples on the GC-MS to remove background and ensure consistency throughout the analysis.

### GC-MS purge and trap method

The volatilome analysis was carried out using an Agilent 7890A GC/5975 single quadrupole system coupled to a Teledyne Tekmar ATOMX XYZ purge and trap system (Palo Alto, CA, USA). The samples were purged at 40 °C for 11 min with nitrogen at 40 ml min^−1^. The trap was kept at room temperature whilst purging and heated to 250 °C for desorption for 2 min. A bake time of 5 min (280 °C) was used in between samples. For the GC-MS measurements, the following conditions were used: 1 µl of the sample was injected in a splitless mode with an injector temperature of 300 °C, a purge flow of 40 ml min^−1^ at 1 min and a flow rate of 1.0 ml min^−1^ helium. Separation was achieved on an Agilent J and W VF5-ms column (30 m×0.25 mm×0.25 mm) (Agilent, Santa Clara, CA) using the following temperature profile: 80 °C for 1 min, 40 °C min^−1^ to 275 °C, 10 °C min^−1^ to 325 °C and 320 °C for 15 min. Ionization employed 70 eV electron ionization, and the mass spectrometer was operated in selected ion mode. In addition to the samples, a C7-C30 saturated alkane standard mixture (Millipore Sigma, USA) was included in the analysis to establish the retention indices.

### Data processing

The identification of VOC confirmation was conducted using MassHunter Qualitative Analysis software (v. B.07.06.2704), and the target compound annotation was performed using the National Institute of Standards and Technology (NIST) mass spectral libraries. Integration was performed using the Agile2 algorithm, with peak detection carried out via chromatogram deconvolution. Compound identification was achieved by matching the resulting mass spectra with entries in the NIST 2017 (v. 2.3) USA spectral database, where compounds with a probability score of 70 or above were annotated to ensure reliable identification. Peaks detected in both the samples and controls, including sterile media, blank vials or water controls, were evaluated using a criterion where the average peak intensity in the samples had to exceed that in the controls by a ratio of greater than 1. Only peaks showing higher abundance in the samples than controls, or those that were undetectable in the controls, were considered for analysis and reporting. Peaks were rejected if they appeared in several controls and only with smaller peak size in EBC or isolate samples.

### Data analysis and visualization

All analyses conducted in this study were primarily qualitative, focusing on matching detected peaks in the EBC samples with those of the microbial cultures. However, the VOC analysis across different growth phases involved quantitative assessment. Unique VOCs detected in EBC samples were mapped using Cytoscape (v. 3.9.1). The plots for data visualization were generated using R (v. 4.4.0).

### Determining volatilome profile of CF isolates under different growth stages

To explore the source of variability in bacterial volatilomes, a separate analysis was carried out to determine the growth patterns of a few CF isolates (PA, *n*=5; SA, *n*=5; SM, *n*=1; RM, *n*=1; AX, *n*=2; *Streptococcus gordonii* (SG), *n*=1) followed by characterization of their volatilome at different growth phases. The growth curves for the CF isolates were obtained according to a method described previously [44]. In brief, the cultures were prepared in BHI media and incubated overnight at 37 °C with continuous shaking at 220 r.p.m. under aerobic conditions. OD_600_ was determined, and cultures were diluted to a final OD_600_ of 0.01 in clear bottom 96-well plates containing fresh BHI media. Growth curves were established using a BioTek Synergy HTX plate reader equipped with Gen5 imaging software (v. 3.10, Agilent). The plates were then incubated at 37 °C under aerobic conditions, with orbital shaking at 237 cycles per minute. OD_600_ measurements were taken every 15 min over a 24 h period to monitor aerobic growth. These measurements were corrected for background, and growth curve analyses were performed using R (v. 4.4.0), utilizing the 'growthcurver' package (v. 0.3.1). The growth curves were used to determine the optimal incubation periods for sampling cultures during the exponential and stationary phases for each CF isolate. Volatile analysis was then performed at these specific time points, with each CF isolate cultured in triplicate. The sample preparation for GC-MS analysis was carried out according to the method described above using 4 ml of filter-sterilized bacterial cultures. Additionally, 9 BHI sterile media controls, 3 Milli-Q water controls and 23 blank vials were prepared and run concurrently with the samples on the GC-MS. VOC analysis across various growth phases was carried out quantitatively. The relative abundances of metabolites at two different time points were visualized using box plots created in R (v. 4.4.0).

## Results

### CB sampling approach to capture bacterial pathogens

Our CB sampling approach was designed to collect an EBC sample whilst simultaneously attempting to culture pathogens from the cough of a pwCF in one collection procedure (Fig. 1). Out of 47 CB samples collected, bacterial cultures were obtained from 18 CB filters (38%); however, only three were positive for SA or PA (6%). Additionally, four cultures were positive for *Streptococcus* species (9%), specifically SG, *Streptococcus anginosus* and *Streptococcus salivarius*, which were included in our analysis. Additionally, 23% of the cultured samples (11) showed non-pathogenic bacterial growth, whilst no bacterial growth was observed in 62% of the CB cultures (29). To enhance the detection of CF pathogen VOCs and improve positivity rates, we employed sputum cultures alongside the CB approach to increase the likelihood of isolating pathogens for volatilomics. Of the 25 sputum samples collected from pwCF, 84% (21 out of 25) of them tested positive for SA, PA or both. Detailed data on the isolates obtained from both CB and sputum samples are provided in Table S1.

### Pathogen-specific VOCs are identified in the EBC of pwCF but infrequently

**Fig. 2.**
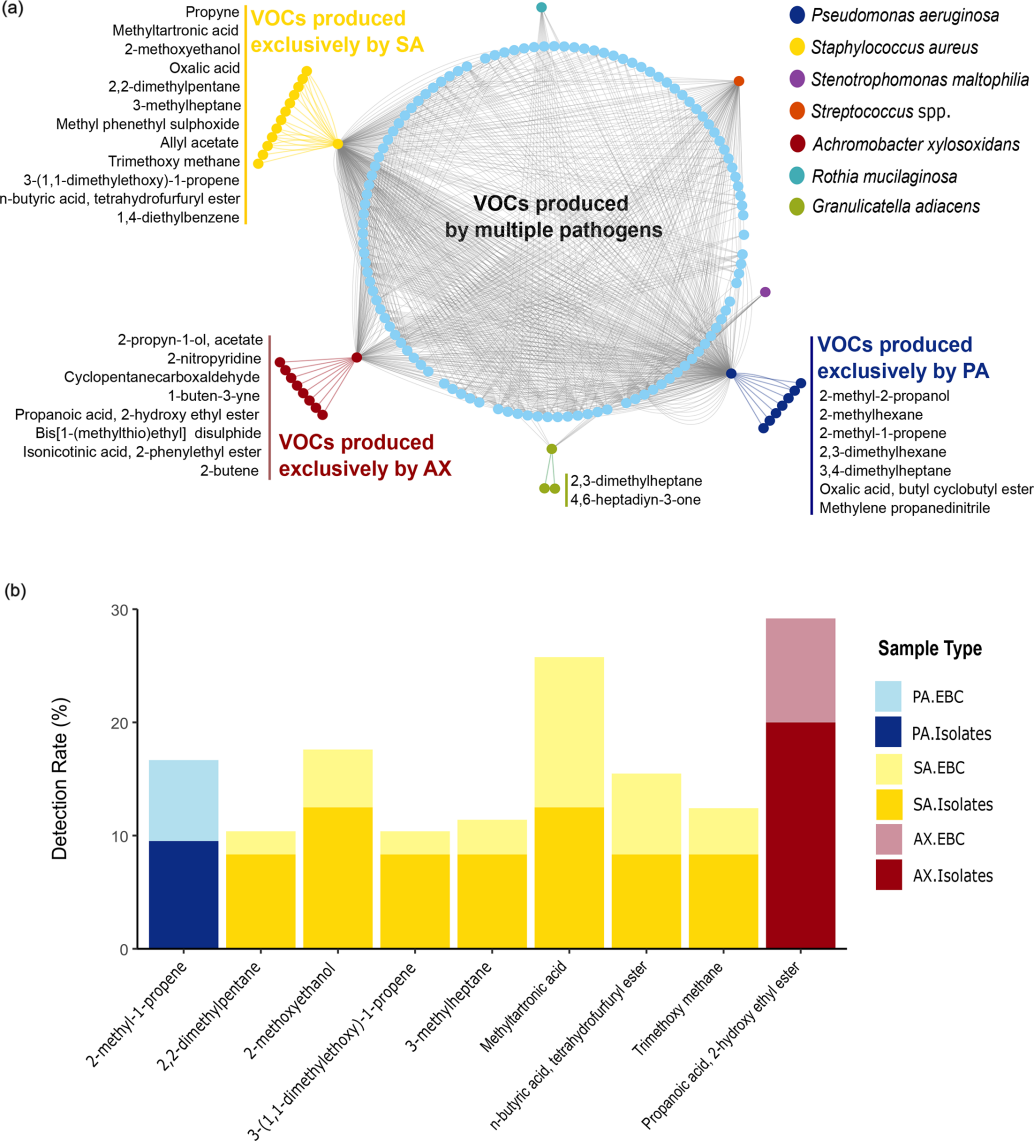
The unique VOCs produced by each CF pathogen detected in the EBC of pwCF. (**a**) Network visualization of the associations between pathogen-specific VOCs and EBC samples from pwCF, generated using Cytoscape v. 3.9.1. The network shows interactions between VOCs detected in EBC that are uniquely produced by CF pathogens including PA, SA, AX, SM, RM, GA and *Streptococcus* spp. The innermost nodes represent VOCs that were emitted by multiple pathogens, whilst the outermost nodes represent VOCs that were exclusively produced by each pathogen and were detected in EBC samples. Each line in the network represents an association between a pathogen, a VOC and its presence in EBC. (b) Pathogen-specific VOC detection rates per isolate and EBC samples. Unique VOCs for each pathogen are displayed, with detection rates based on the percentage of isolates or EBC samples in which they were present. Detection rates were calculated as a percentage of total isolates for each species: PA (*n*=21), SA (*n*=24), AX (*n*=10) and EBC samples (*n*=98). VOCs detected in at least two isolates or EBC samples are shown.

We analysed the volatilome profiles of 7 different microbial species (65 isolates) and 98 EBC samples to identify VOCs present in the EBC that were exclusively produced by the isolates of PA, SA, AX, SM, RM, GA or *Streptococcus* spp. (microbial VOC must be present in at least two EBC samples or isolates). Seven, twelve and eight VOCs produced exclusively by PA, SA and AX isolates, respectively, were also detected in EBC (Fig. 2a). Methyltartronic acid and allyl acetate were the most frequently detected SA-specific VOCs in EBC, found in 13 and 12 out of 98 samples, respectively. Among these, 46% of methyltartronic acid-positive samples and 67% of allyl acetate-positive samples were confirmed to have SA by clinical culture. Additionally, 2-methyl-1-propene was detected in seven EBC samples, of which two tested positive for PA. In addition to the primary CF pathogens, GA was found to produce unique VOCs detectable in the EBC of pwCF, including 2,3-dimethylheptane (six EBCs) and 4,6-heptadiyn-3-one (two EBCs). However, we did not detect any specific VOCs for SM, RM or *Streptococcus* spp. strains in our collection.

Although we were able to match unique VOCs from CF pathogens of interest with those in the EBC, the incidence of detection and consistency across isolates was low (Fig. 2b). For SA, 12 unique VOCs were identified in the EBC, but only 2-methoxyethanol and methyltartronic acid were identified in at least 12% of the isolates, with detection rates of 5 and 13% in EBC samples, respectively. Additionally, five other VOCs exclusively produced by SA – 2,2-dimethylpentane, 3-(1,1-dimethylethoxy)-1-propene, 3-methylheptane, tetrahydrofurfuryl ester n-butyric acid and trimethoxy methane – were detected in just 8% of isolates. 2-Methyl-1-propene, specific to PA, was detected in 7% of EBC samples but in only 10% of the isolates tested. 2-Hydroxy ethyl ester propanoic acid, unique to AX, was detected in 20% of isolates and 9% of the EBC. These findings show that VOCs unique to each pathogen of interest were present in the EBC of pwCF, making them potentially valuable diagnostic markers, but they were rare across strains of the same species. Due to this unexpected strain level variability, we expanded our analysis to identify VOCs common across several CF pathogens, treating them as collective markers of bacterial infection, and then explored some of the sources of this observed variability.

### Certain VOCs show potential as collective markers of bacterial infection

**Fig. 3.**
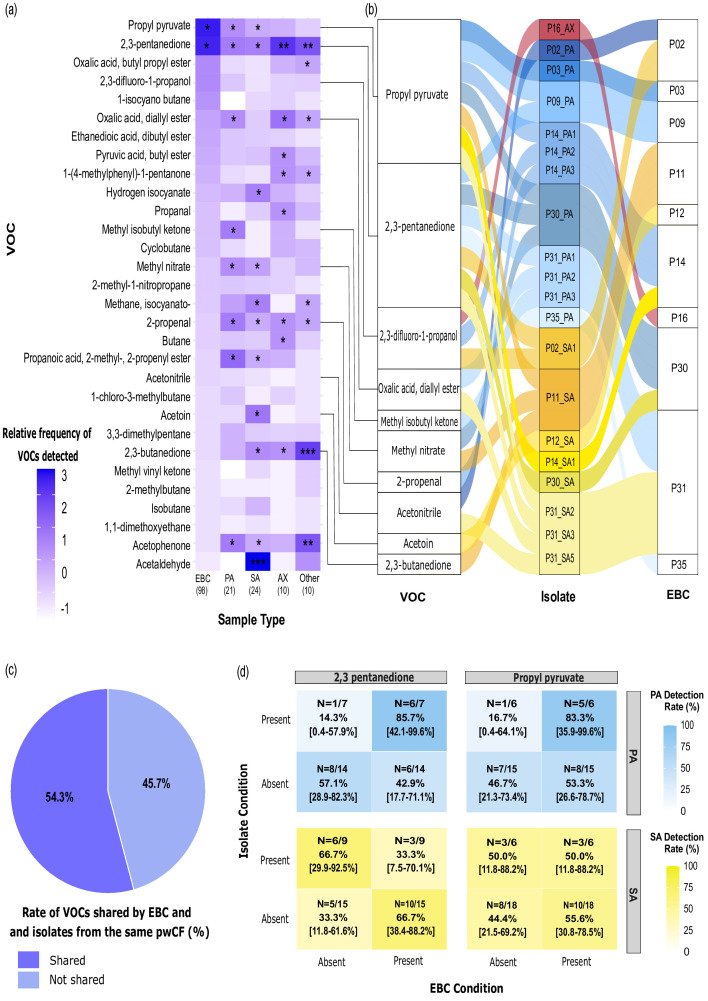
Overview of VOCs detected in EBC and their associations with CF pathogens. (**a**) Heatmap illustrating the relative frequency of VOCs across various sample types highlighted with their corresponding *Z*-scores accounting for different sample sizes of each group. The colour gradient in the heatmap represents *Z*-scores, indicating how the relative frequency of each VOC in a sample type deviates from the overall mean. VOCs are arranged based on their detection frequency in EBC, with the most frequently identified VOCs at the top and less frequent ones at the bottom. Asterisks indicate the detection frequency of each VOC within sample types as follows: *25–49.99%, **50–74.99% and ***>75%. VOCs with a detection frequency below 25% are not marked with an asterisk, and samples without any VOC detection are not coloured. ‘Other’ CF pathogens include SM, RM, GA and *Streptococcus* spp. (**b**) Alluvial plot illustrating the connections between VOCs detected in isolates and the EBC of the same pwCF. Isolates are colour-coded based on the pathogens: yellow for SA, blue for PA and maroon for AX, showing the specific associations between pathogens and VOCs. (**c**) The percentage of EBC samples that shared volatiles with isolates from the same pwCF, calculated based on the number of EBC samples containing VOCs shared with isolates of key CF pathogens, including PA, SA and AX (*n*=19), relative to the total number of EBC samples matched to an isolate from the same pwCF (*n*=35). (**d**) Confusion matrix heatmap of VOC detection accuracy in EBC and corresponding isolates from the same pwCF. The heatmap displays detection accuracy for key VOCs, including 2,3-pentanedione and propyl pyruvate across different conditions. Rows indicate compound presence or absence in isolates, whilst columns represent presence or absence in EBC samples, with colour gradients for PA (blue) and SA (yellow). Detection rates are shown as the percentage of samples with matching VOCs in both isolates and EBC, with CIs included to address variability due to sample size differences.

To identify VOCs more broadly indicative of bacterial infection, we initially screened those most frequently detected in the EBC of pwCF against the volatilomes of diverse CF pathogens (Fig. 3a). Detection frequencies of the VOCs were standardized across bacterial species and EBC samples using *Z*-scores to ensure comparability given the varying sample sizes. Among the frequently detected VOCs in EBC, 30 compounds were produced by multiple CF pathogens. Sixteen VOCs were identified as common between EBC and all four CF pathogen groups (PA, SA, AX and others; SM, RM, GA and *Streptococcus* spp.). The most frequently detected shared microbial VOCs in EBC were propyl pyruvate (43%) and 2,3-pentanedione (42%). Propyl pyruvate was detected in isolates of SA (25%), PA (29%), AX (20%) and *Streptococcus* spp. (14%), whereas 2,3-pentanedione was detected in isolates of SA (42%), PA (33%), AX (60%) and *Streptococcus* spp. (57%). Specifically, 2,3-pentanedione has previously been identified as a core volatile metabolite produced by clinical isolates of PA [38]. Methyl nitrate and 2-methyl-2-propenyl ester propanoic acid were detected across all species tested but were most predominant in PA (29 and 38%) and SA (29 and 25%), respectively. Methyl isobutyl ketone, a compound previously identified as a core VOC from PA [38], was detected in PA (33%) and AX (20%) isolates. Acetoin, commonly produced by fermentative bacteria [45,46], such as SA and *Streptococcus* spp., was detected in multiple CF pathogens but was particularly enriched in SA isolates (46%). Acetaldehyde, a VOC commonly produced by SA [47,48], was detected in 84% of the SA isolates. However, its presence in EBC samples was markedly lower, with only 2% of EBC samples showing acetaldehyde. Butane, propanal and butyl ester pyruvic acid were detected in all isolates, with a predominance in AX (30%). 2,3-Butanedione, a compound previously associated with *Streptococcus* spp. [49] and detected in CF breath, was enriched in the *Streptococcus* isolates used in our study (100%), including SG, *S. anginosus*, *S. salivarius* and *Streptococcus parasanguinis*. However, this compound was also detected in PA (19%), SA (38%), AX (30%) and RM. In addition to the VOCs discussed above, we identified several compounds, including 1-chloro-3-methyl-butane, 2-butanone and methyl vinyl ketone in EBC that were produced by multiple CF pathogens and had previously been reported as putative volatile metabolites of PA clinical isolates [38]. The clinical infection profiles associated with frequently detected VOCs in the EBC of pwCF are shown in Fig. S1. Further, the diversity analysis, including Shannon index, evenness, and richness, of the volatilome profile in EBC samples from pwCF under different clinical conditions showed no significant difference between pwCF who tested positive or negative for PA and SA at the time of sample collection (Fig. S2).

### Connections between VOCs of isolates and EBC from the same pwCF

Using both CB filters and sputum cultures, we were able to obtain a substantial proportion of isolates matched with EBC samples from the same patient: 71% (17 out of 24) of SA isolates, 76% (16 out of 21) of PA isolates, 60% (6 out of 10) of AX isolates and 100% (all 7) of *Streptococcus* spp. It is also worth noting that 35 out of the 98 EBC samples (36%) had at least one matching isolate from the same individual. Thus, we conducted a comparative analysis of VOC profiles between isolates of SA, PA and AX and their corresponding EBC samples from the same pwCF to see if positivity rates would increase compared to unmatched isolates and EBC. Notable compounds such as propyl pyruvate, 2,3-pentanedione, 2,3-difluoro-1-propanol, oxalic acid diallyl ester, methyl isobutyl ketone, methyl nitrate, 2-propenal, acetonitrile, acetoin and 2,3-butanedione were detected in both the isolates and EBC samples from the same individuals (Fig. 3b). We found that 19 out of the 35 (54%) paired microbial and EBC samples contained matched volatiles, whilst 46% of the EBC samples did not match any volatiles detected in their corresponding isolates (Fig. 3c). Notably, 2,3-pentanedione and propyl pyruvate showed the highest correspondence between EBC samples and isolates from the same pwCF (Fig. 3b).

To determine whether including isolates from the same individuals could enhance detection rates for CF pathogens, particularly PA and SA, we conducted a diagnostic accuracy analysis using a confusion matrix (Fig. 3d). This approach allowed us to systematically assess the true positive (TP), true negative, false positive and false negative rates for key VOCs in EBC. Our analysis revealed that the TP detection rates for 2,3-pentanedione and propyl pyruvate were higher in PA isolates, with rates of 86% (CI: 42.1–99.6%) and 83% (CI: 35.9–99.6%), respectively. This means that 2,3-pentanedione and propyl pyruvate, produced by the PA isolates, were 86 and 83% accurately identified in the corresponding EBC samples, respectively. In contrast, the TP rates for SA isolates were found to be 33% (CI; 7.5–70.1%) for 2,3-pentanedione and 50% (CI; 11.8–88.2%) for propyl pyruvate. Thus, comparing VOC detection between isolates and EBC samples from the same pwCF demonstrated higher TP rates than across the population, particularly for PA. This suggests that acquiring an isolate from each pwCF may aid VOC detection in the clinical context.

### Volatilomes vary across CF isolates of the same species and growth stages

**Fig. 4.**
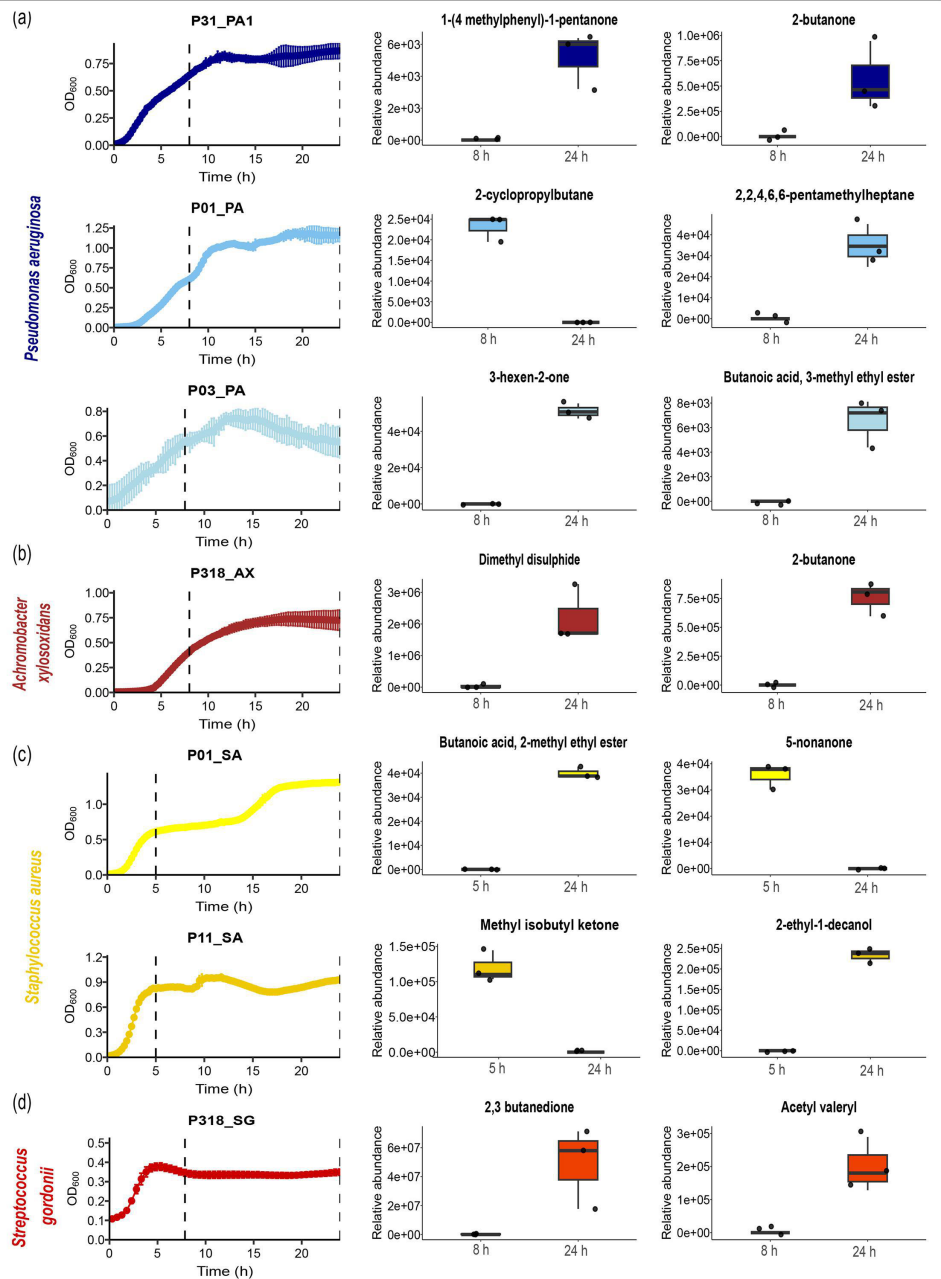
Variation in VOC production by different strains of CF isolates over 24 h across various growth phases. Growth curves are shown for (a) PA, (**b**) AX, (**c**) SA and (d) SG, alongside the VOCs consistently detected across all three replicates for each isolate at specific time points during the exponential and stationary growth phases.

Although our approach to mapping VOCs from CF clinical isolates to the EBC of pwCF was able to identify species-specific and broader metabolite indicators of airway infection, the consistency across subjects and within bacterial isolates of the same species was unexpectedly low. Thus, we explored the variation in volatiles of interest during different growth phases of bacterial isolates to better understand the timing of their production. Fig. 4 illustrates the variation observed in the volatilome of CF isolates within each species during late exponential and stationary phases. The objective of this analysis was to optimize methods for assessing the volatilome of CF isolates by examining them across different growth stages, including late exponential and stationary phases providing a comprehensive understanding of their metabolic activity and potential diagnostic markers. In this analysis, only VOCs that were consistently present in all three replicates for each isolate were included. Based on the growth curves obtained for each CF isolate, the optimal time points for capturing VOCs during exponential and stationary phases were identified. For PA (Fig. 4a), AX (Fig. 4b) and *Streptococcus* spp. (Fig. 4d) isolates, these phases were determined to be at 8 and 24 h, respectively. However, for SA isolates (Fig. 4c), the exponential and stationary phases were selected to be at 5 and 24 h, respectively, due to their faster growth. Understanding these growth dynamics allowed us to select appropriate time points for VOC analysis across different growth stages of CF isolates.

We found for all isolates and numerous volatiles that the growth stage altered their production. In PA isolates, VOCs such as 3-hexen-2-one, 2,2,4,6,6-pentamethylheptane and 3-methyl ethyl ester butanoic acid were present at 24 h but absent at 8 h. In contrast, 2-cyclopropylbutane was detected at 8 h but not at 24 h (Fig. 4a). For AX isolate, dimethyl disulphide and 2-butanone were detected at 24 h but absent at 8 h (Fig. 4b). In SA isolates, 2-methyl ethyl ester butanoic acid and 2-ethyl-1-decanol appeared only at 24 h, whereas methyl isobutyl ketone and 5-nonanone were detected at 5 h but not at 24 h (Fig. 4c). Similarly, in *Streptococcus* spp., 2,3-butanedione and acetyl valeryl were found at 24 h but absent at 8 h (Fig. 4d).

Importantly, certain VOCs detected were not uniformly present across all isolates of a given species; rather, their production was specific to isolates and varied significantly between different growth phases. This underscores the variability in VOC profiles among different isolates and emphasizes the need for a comprehensive approach when studying the volatilomes of CF pathogens. This phase-dependent variation may further complicate the identification of consistent biomarkers and highlight the dynamic nature of microbial metabolism under different growth stages.

## Discussion

Our study aimed to identify unique microbial volatile metabolites produced by CF pathogens that could serve as markers of infection in CF airways. Identifying VOCs indicative of certain pathogens in the airways could lead to the development of early detection and tracking approaches for CF clinical diagnostics. This is especially important in the current era of highly effective CFTR modulator therapies, which have drastically reduced the availability of the gold-standard microbiological diagnostic sample of sputum [15,17]. Without sputum production, it becomes particularly challenging to monitor infections of pwCF, which are persisting in the new CFTR modulator era [15,18, 19]. Furthermore, a non-invasive approach to monitoring infections, such as the CB method developed here, would be especially advantageous for timely and targeted clinical interventions. In its most basic goal, of identifying microbial VOCs in the EBC of pwCF both unique to certain CF pathogens and found more broadly among species, our study was successful; however, the consistency and frequency of production of these metabolites were low, requiring more optimization and a deeper understanding of the growth characteristics that drive the production of metabolites of interest.

In our initial analysis, we identified seven specific VOCs linked to SA including methyltartronic acid, 2-methoxyethanol, 2,2-dimethylpentane, 3-(1,1-dimethylethoxy)-1-propene, 3-methylheptane, tetrahydrofurfuryl ester n-butyric acid and trimethoxy methane in the EBC of pwCF. Additionally, 2-methyl-1-propene unique to PA and 2-hydroxy ethyl ester propanoic acid specific to AX were detected in the EBC. However, these species-unique compounds exhibited low detection rates across isolates and in the EBC, underscoring significant variability in the volatilome profile. Though strain-specific differences in bacterial volatilomes remain relatively underexplored, existing studies have shown distinct variability across strains. For instance, a previous study demonstrated the ability to differentiate between multiple strains of PA in the breath of infected mice, highlighting that unique VOCs could serve as markers for specific bacterial strains in infectious disease models [50].

Whilst prior research has often focused on specific biomarkers produced by individual CF pathogens such as PA [23,38, 40, 51,53] or SA [25,39, 53], few studies have reported shared markers produced by multiple CF pathogens [54,55]. Our study fills this gap by identifying collective infection markers, which may be more broadly indicative of CF airway infection in the era of ETI. Notably, VOCs such as 2,3-pentanedione, propyl pyruvate, methyl isobutyl ketone, acetonitrile, acetoin and 2,3-butanedione were shared microbial volatiles frequently detected in EBC from pwCF. Among these, acetoin is a commonly produced VOC by fermentative bacteria like SA and *Streptococcus* spp. and was detected across several CF pathogens in our study, with notable enrichment in SA isolates (46%). This is consistent with a previous study indicating that acetoin production is associated with the fermentation pathways of bacteria commonly found in the CF lung, contributing to the overall metabolic profile of these pathogens [45,46]. 2,3-Pentanedione emerged as one of the most prominent VOCs detected across pathogens analysed in this study and was detected in 42% of the EBC samples with an enhancement of positivity rate (86%) when examining isolates from the same pwCF. Previously recognized as a core volatile metabolite produced by clinical isolates of PA [38], 2,3-pentanedione has also been reported as a by-product of aa synthesis during the fermentation of alcoholic beverages, particularly from isoleucine [56,57]. This suggests that, similar to the metabolic pathway identified for diacetyl (2,3-butanedione) [49], 2,3-pentanedione could be generated through analogous processes in the CF airways, which may explain its detection in EBC. Thus, 2,3-pentanedione holds promise as a valuable marker for detecting airway infection more generally, even when the causative pathogen cannot be readily identified. Further exploration of 2,3-pentanedione production in EBC and other breath samples from pwCF is needed to explore its potential as an infection biomarker, especially in those on modulator therapy.

Despite its fundamental success in matching microbial VOCs to those in the EBC from pwCF, our study encountered numerous challenges, particularly the low incidence of detection of these infection-related VOCs, even in patients known to be infected with these pathogens. Surprisingly, we found that many VOCs of interest matching the EBC volatilomes were only detected from a few isolates of each species (e.g. SA and PA), emphasizing that there is significant variability in the volatilomes of different airway pathogens. A previous study conducted a comparative analysis of the volatilomes of 24 PA clinical isolates using untargeted analysis and revealed significant metabolomic diversity, with only 18% of the pan-volatilome being conserved across these isolates [38]. This demonstrates high levels of volatilome variability even in a relatively small sample of clinical isolates. This is mirrored in analyses of PA pangenomes, where sequencing of over 1300 PA isolates revealed that only 1% of the pan-genome corresponding to the core genome is being conserved across all isolates [58]. In a previous study, temporal volatilomic analysis of multiple strains from PA and SA revealed notable differences in the emission of specific compounds across the strains studied [47], which varied by stage, with some molecules produced only during the exponential growth phase and others only during the lag phase. Another possible explanation for the low matching rate between microbial VOCs and those from EBC is the fact that the majority of subjects in our study were currently taking modulators (83%). These drugs have significantly reduced the infectious load of bacterial pathogens in the airways [15,18, 19], which would intuitively lower the amount of their volatiles. Studies of microbial volatiles in EBC from people not on modulators may help demonstrate this, but these individuals are becoming rarer as more pwCF are becoming eligible for modulator treatment. Regardless, this study showed that the CB and EBC detection method can identify microbial molecules in the airways of pwCF, but identification of VOCs consistently produced across pathogens of interest is paramount to diagnostic utility.

Whilst our study provides valuable insights, we acknowledge several limitations. Whilst our study primarily employed a qualitative approach, future studies should aim to include a larger cohort to enhance statistical power and further validate our findings. Expanding the sample size and incorporating additional clinical and microbial data will strengthen the robustness of VOC detection and improve the generalizability of the results. Further, the freezing of EBC samples, though necessary for logistical reasons, may have led to some loss of VOCs. Whilst previous studies suggest that certain VOCs remain detectable after freezing, differential loss remains a potential limitation. Freezing may preserve some reactive or unstable compounds, but it could also allow for the sublimation of volatiles into the headspace, which would be lost upon opening the storage container [41]. Additionally, whilst the BHI medium is often used in microbial VOC analysis [38,40], it may not fully replicate the CF lung environment. Because different media formulations can drastically affect VOC production from microbes [59], future work should focus on using experimental conditions that better simulate the CF airway microbiome and explore the effects of different media formulations. This could be achieved by growing CF pathogens in artificial sputum media that mimic the conditions of the CF lung, particularly under the influence of ETI therapy, which will provide a more physiologically relevant model for further examining the VOC profiles of PA and SA in CF.

This study developed an innovative dual sampling approach known as CB, as a non-invasive method to identify and study airway bacteria from pwCF who can no longer produce sputum due to the effects of ETI. This method can isolate CF pathogens in those that cannot produce sputum through culturing the CB filters and identify volatiles in the simultaneously collected EBC. However, detection and isolation rates were low, which could be driven by therapeutic (ie. modulators, antibiotics or others) and microbial physiological factors requiring further study and optimization. Perhaps the most promising application of CB is the ability to detect infections more generally, which may be particularly relevant in the era of ETI where infectious load is generally lower and harder to detect, and the gold-standard clinical sample of sputum is not readily available. Beyond its intended use as a novel diagnostic, the CB approach can also shed light on the diverse volatile metabolites produced in the human airway, providing insight into the host and microbial physiology occurring during polymicrobial infections.

## Supplementary material

10.1099/mic.0.001536 Uncited Supplementary Material 1.

## Abbreviations

AX: *Achromobacter xylosoxidans*
BHI: brain heart infusion
CB: cough breath
CF: cystic fibrosis
CFTR: cystic fibrosis transmembrane conductance regulator
EBC: exhaled breath condensate
ETI: elexacaftor-tezacaftor-ivacaftor
GA: *Granulicatella adiacens*
NIST: National Institute of Standards and Technology
OD_600_: OD at 600 nm
PA: *Pseudomonas aeruginosa*
PTFE: polytetrafluoroethylene
pwCF: people with cystic fibrosis
RM: *Rothia mucilaginosa*
SA: *Staphylococcus aureus*
SG: *Streptococcus gordonii*
SM: *Stenotrophomonas maltophilia*
TP: true positive
UCSD: University of California San Diego
VOCs: volatile organic compounds

## References

[1] Zang X, Monge ME, Gaul DA, McCarty NA, Stecenko A (2020). Early detection of cystic fibrosis acute pulmonary exacerbations by exhaled breath condensate metabolomics. J Proteome Res.

[2] Mahboubi MA, Carmody LA, Foster BK, Kalikin LM, VanDevanter DR (2016). Culture-based and culture-independent bacteriologic analysis of cystic fibrosis respiratory specimens. J Clin Microbiol.

[3] Goss CH, Burns JL (2007). Exacerbations in cystic fibrosis: 1. epidemiology and pathogenesis. Thorax.

[4] Hauser AR, Jain M, Bar-Meir M, McColley SA (2011). Clinical significance of microbial infection and adaptation in cystic fibrosis. Clin Microbiol Rev.

[5] Rogers GB, Carroll MP, Serisier DJ, Hockey PM, Jones G (2004). Characterization of bacterial community diversity in cystic fibrosis lung infections by use of 16S ribosomal DNA terminal restriction fragment length polymorphism profiling. J Clin Microbiol.

[6] Cuthbertson L, Walker AW, Oliver AE, Rogers GB, Rivett DW (2020). Lung function and microbiota diversity in cystic fibrosis. Microbiome.

[7] Zemanick ET, Hoffman LR (2016). Cystic fibrosis: microbiology and host response. Pediatr Clin North Am.

[8] Fenn D, Abdel-Aziz MI, Brinkman P, Kos R, Neerincx AH (2022). Comparison of microbial composition of cough swabs and sputum for pathogen detection in patients with cystic fibrosis. J Cyst Fibros.

[9] Seidler D, Griffin M, Nymon A, Koeppen K, Ashare A (2016). Throat swabs and sputum culture as predictors of *P. aeruginosa* or *S. aureus* lung colonization in adult cystic fibrosis patients. PLoS One.

[10] Lu J, Carmody LA, Opron K, Simon RH, Kalikin LM (2020). Parallel analysis of cystic fibrosis sputum and saliva reveals overlapping communities and an opportunity for sample decontamination. mSystems.

[11] Schultz A, Caudri D (2018). Cough swabs less useful but induced sputum very useful in symptomatic older children with cystic fibrosis. Lancet Respir Med.

[12] Sosinski LM, H CM, Neugebauer KA, Ghuneim LAJ, Guzior DV (2022). A restructuring of microbiome niche space is associated with elexacaftor-tezacaftor-ivacaftor therapy in the cystic fibrosis lung. J Cyst Fibros.

[13] Nichols DP, Paynter AC, Heltshe SL, Donaldson SH, Frederick CA (2022). Clinical effectiveness of elexacaftor/tezacaftor/ivacaftor in people with cystic fibrosis: a clinical trial. Am J Respir Crit Care Med.

[14] Sutharsan S, Dillenhoefer S, Welsner M, Stehling F, Brinkmann F (2023). Impact of elexacaftor/tezacaftor/ivacaftor on lung function, nutritional status, pulmonary exacerbation frequency and sweat chloride in people with cystic fibrosis: real-world evidence from the German CF Registry. Lancet Reg Heal Eur.

[15] Nichols DP, Morgan SJ, Skalland M, Vo AT, Van Dalfsen JM (2023). Pharmacologic improvement of CFTR function rapidly decreases sputum pathogen density, but lung infections generally persist. J Clin Invest.

[16] Schaupp L, Addante A, Völler M, Fentker K, Kuppe A (2023). Longitudinal effects of elexacaftor/tezacaftor/ivacaftor on sputum viscoelastic properties, airway infection and inflammation in patients with cystic fibrosis. Eur Respir J.

[17] Kos R, Neerincx AH, Fenn DW, Brinkman P, Lub R (2022). Real-life efficacy and safety of elexacaftor/tezacaftor/ivacaftor on severe cystic fibrosis lung disease patients. Pharmacol Res Perspect.

[18] Martin C, Guzior DV, Gonzalez CT, Okros M, Mielke J (2023). Longitudinal microbial and molecular dynamics in the cystic fibrosis lung after elexacaftor-tezacaftor-ivacaftor therapy. Res Sq.

[19] Tunney MM, Wark P (2023). Long-term therapy with elexacaftor/tezacaftor/ivacaftor (ETI) in cystic fibrosis: improved clinical outcomes but infection and inflammation persist. Eur Respir J.

[20] Rosenfeld M, Emerson J, Accurso F, Armstrong D, Castile R (1999). Diagnostic accuracy of oropharyngeal cultures in infants and young children with cystic fibrosis. Pediatr Pulmonol.

[21] Zemanick ET, Wagner BD, Robertson CE, Stevens MJ, Szefler SJ (2015). Assessment of airway microbiota and inflammation in cystic fibrosis using multiple sampling methods. Ann Am Thorac Soc.

[22] Ahmed B, Bush A, Davies JC (2014). How to use: bacterial cultures in diagnosing lower respiratory tract infections in cystic fibrosis. *Arch Dis Child Educ Pract Ed*.

[23] Kos R, Brinkman P, Neerincx AH, Paff T, Gerritsen MG (2022). Targeted exhaled breath analysis for detection of *Pseudomonas aeruginosa* in cystic fibrosis patients. J Cyst Fibros.

[24] Kramer R, Sauer-Heilborn A, Welte T, Guzman CA, Höfle MG (2015). A rapid method for breath analysis in cystic fibrosis patients. Eur J Clin Microbiol Infect Dis.

[25] Neerincx AH, Geurts BP, van Loon J, Tiemes V, Jansen JJ (2016). Detection of *Staphylococcus aureus* in cystic fibrosis patients using breath VOC profiles. J Breath Res.

[26] Bos LDJ, Meinardi S, Blake D, Whiteson K (2016). Bacteria in the airways of patients with cystic fibrosis are genetically capable of producing VOCs in breath. J Breath Res.

[27] Gaisl T, Bregy L, Stebler N, Gaugg MT, Bruderer T (2018). Real-time exhaled breath analysis in patients with cystic fibrosis and controls. J Breath Res.

[28] van Aerde KJ, Leegstraten A, van den Kieboom CH, Merkus P, Sintnicolaas C (2022). Non-invasive diagnostics of pathogenic bacteria using a breath sampler in children with cystic fibrosis. J Breath Res.

[29] Boots AW, Bos LD, van der Schee MP, van Schooten F-J, Sterk PJ (2015). Exhaled molecular fingerprinting in diagnosis and monitoring: validating volatile promises. Trends Mol Med.

[30] Horváth I, Barnes PJ, Loukides S, Sterk PJ, Högman M (2017). A european respiratory society technical standard: exhaled biomarkers in lung disease. Eur Respir J.

[31] Ghosh C, Leon A, Koshy S, Aloum O, Al-Jabawi Y (2021). Breath-based diagnosis of infectious diseases: a review of the current landscape. Clin Lab Med.

[32] Lechner M, Fille M, Hausdorfer J, Dierich MP, Rieder J (2005). Diagnosis of bacteria *in vitro* by mass spectrometric fingerprinting: a pilot study. Curr Microbiol.

[33] Nizio KD, Perrault KA, Troobnikoff AN, Ueland M, Shoma S (2016). *In vitro* volatile organic compound profiling using GC×GC-TOFMS to differentiate bacteria associated with lung infections: a proof-of-concept study. J Breath Res.

[34] Tsou P-H, Lin Z-L, Pan Y-C, Yang H-C, Chang C-J (2021). Exploring volatile organic compounds in breath for high-accuracy prediction of lung cancer. Cancers.

[35] Wang P, Huang Q, Meng S, Mu T, Liu Z (2022). Identification of lung cancer breath biomarkers based on perioperative breathomics testing: a prospective observational study. EClinicalMedicine.

[36] Zou Y, Hu Y, Jiang Z, Chen Y, Zhou Y (2022). Exhaled metabolic markers and relevant dysregulated pathways of lung cancer: a pilot study. Ann Med.

[37] Jia Z, Thavasi V, Venkatesan T, Lee P (2023). Breath analysis for lung cancer early detection—a clinical study. Metabolites.

[38] Bean HD, Rees CA, Hill JE (2016). Comparative analysis of the volatile metabolomes of *Pseudomonas aeruginosa* clinical isolates. J Breath Res.

[39] Jenkins CL, Bean HD (2023). Current limitations of staph infection diagnostics, and the role of VOCs in achieving culture-independent detection. Pathogens.

[40] Davis TJ, Karanjia AV, Bhebhe CN, West SB, Richardson M (2020). *Pseudomonas aeruginosa* volatilome characteristics and adaptations in chronic cystic fibrosis lung infections. Sphere.

[41] Davis MD, Montpetit AJ (2018). Exhaled breath condensate: an update. Immunol Allergy Clin North Am.

[42] Hunt J (2007). Exhaled breath condensate: an overview. Immunol Allergy Clin North Am.

[43] van Mastrigt E, de Jongste JC, Pijnenburg MW (2015). The analysis of volatile organic compounds in exhaled breath and biomarkers in exhaled breath condensate in children - clinical tools or scientific toys?. Clin Exp Allergy.

[44] Guzior DV, Okros M, Shivel M, Armwald B, Bridges C (2024). Bile salt hydrolase acyltransferase activity expands bile acid diversity. *Nature*.

[45] Fitzgerald S, Holland L, Morrin A (2021). An investigation of stability and species and strain-level specificity in bacterial volatilomes. Front Microbiol.

[46] Zhou C, Fey PD (2020). The acid response network of *Staphylococcus aureus*. Curr Opin Microbiol.

[47] O’Hara M, Mayhew CA (2009). A preliminary comparison of volatile organic compounds in the headspace of cultures of *Staphylococcus aureus* grown in nutrient, dextrose and brain heart bovine broths measured using a proton transfer reaction mass spectrometer. J Breath Res.

[48] Chippendale TWE, Gilchrist FJ, Španěl P, Alcock A, Lenney W (2014). Quantification by SIFT-MS of volatile compounds emitted by *in vitro* cultures of *S. aureus*, *S. pneumoniae*, and *H. influenzae* isolated from patients with respiratory diseases. Anal Methods.

[49] Whiteson KL, Meinardi S, Lim YW, Schmieder R, Maughan H (2014). Breath gas metabolites and bacterial metagenomes from cystic fibrosis airways indicate active PH neutral 2,3-butanedione fermentation. ISME J.

[50] Purcaro G, Nasir M, Franchina FA, Rees CA, Aliyeva M (2019). Breath metabolome of mice infected with *Pseudomonas aeruginosa*. Metabolomics.

[51] Scott-Thomas AJ, Syhre M, Pattemore PK, Epton M, Laing R (2010). 2-Aminoacetophenone as a potential breath biomarker for *Pseudomonas aeruginosa* in the cystic fibrosis lung. BMC Pulm Med.

[52] Gilchrist FJ, Belcher J, Jones AM, Smith D, Smyth AR (2015). Exhaled breath hydrogen cyanide as a marker of early *Pseudomonas aeruginosa* infection in children with cystic fibrosis. ERJ Open Res.

[53] Nasir M, Bean HD, Smolinska A, Rees CA, Zemanick ET (2018). Volatile molecules from bronchoalveolar lavage fluid can "rule-in" *Pseudomonas aeruginosa* and "rule-out" *Staphylococcus aureus* infections in cystic fibrosis patients. Sci Rep.

[54] Zhu J, Bean HD, Kuo YM, Hill JE (2010). Fast detection of volatile organic compounds from bacterial cultures by secondary electrospray ionization-mass spectrometry. J Clin Microbiol.

[55] Mustafina M, Silantyev A, Krasovskiy S, Chernyak A, Naumenko Z (2024). Identification of exhaled metabolites correlated with respiratory function and clinical features in adult patients with cystic fibrosis by real-time proton mass spectrometry. Biomolecules.

[56] Stewart GG (2017). The production of secondary metabolites with flavour potential during brewing and distilling wort fermentations. Fermentation.

[57] Krogerus K, Gibson BR (2013). Influence of valine and other amino acids on total diacetyl and 2,3-pentanedione levels during fermentation of brewer’s wort. Appl Microbiol Biotechnol.

[58] Freschi L, Vincent AT, Jeukens J, Emond-Rheault JG, Kukavica-Ibrulj I (2019). The *Pseudomonas aeruginosa* pan-genome provides new insights on its population structure, horizontal gene transfer, and pathogenicity. Genome Biol Evol.

[59] Jenkins CL, Bean HD (2020). Dependence of the staphylococcal volatilome composition on microbial nutrition. Metabolites.

