# Supplementary material for: Linking volatile metabolites from bacterial pathogens to exhaled breath condensate of people with cystic fibrosis
Source: Microbiology (Reading). 2025 Feb 20;171(2):001536. doi: 10.1099/mic.0.001536 (PMC12282304; doi:10.1099/mic.0.001536)
Supplement: Uncited Supplementary Material 1. [file mic-171-01536-s001.pdf]

Linking Volatile Metabolites from Bacterial Pathogens to Exhaled Breath Condensate of People with Cystic Fibrosis

P. Hansani Karunarathne[1] Christopher Bridges[1] Lacy Remisoski[1], Madisen Crane[1], Claudia Soria Casanova[1], Samantha N. Kinne[2], Alicia L. Castillo Bahena[2], Marissa Gil[3], Lienwil Padillo[3], Gabriel Querido[3], Jenna Mielke[3], Marc McClelland[2], Doug Conrad[3], and Robert A. Quinn[1]

[1]Department of Biochemistry and Molecular Biology, Michigan State University, East Lansing, MI, USA.

[2] Corewell Health, Grand Rapids, MI, USA.

[3] Department of Medicine, University of California San Diego, La Jolla, CA, USA.

Supplementary results

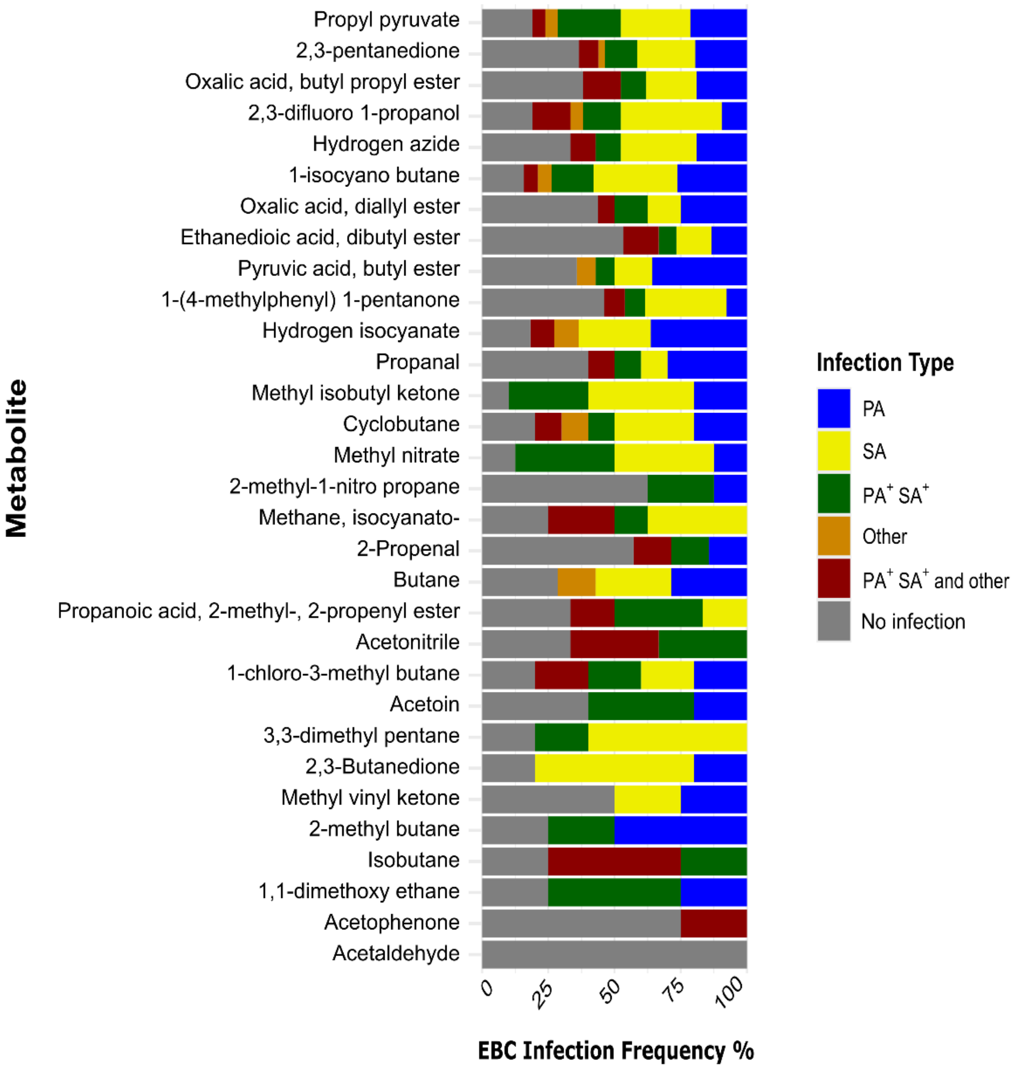

Figure S1. Clinical infection profiles linked to frequently detected VOCs in the EBC of pwCF. Distribution of clinical infection types associated with each VOC identified in the EBC samples from pwCF. The distribution is calculated based on the total number of EBC samples where each VOC was detected under each infection category; *P. aeruginosa* (PA), *S. aureus* (SA). #Other infection types include one or more of the following species: *A. xylosoxidans*, *G. adiacens*, *S. maltophilia*, *R. mucilaginosa*, and *Streptococcus* spp.

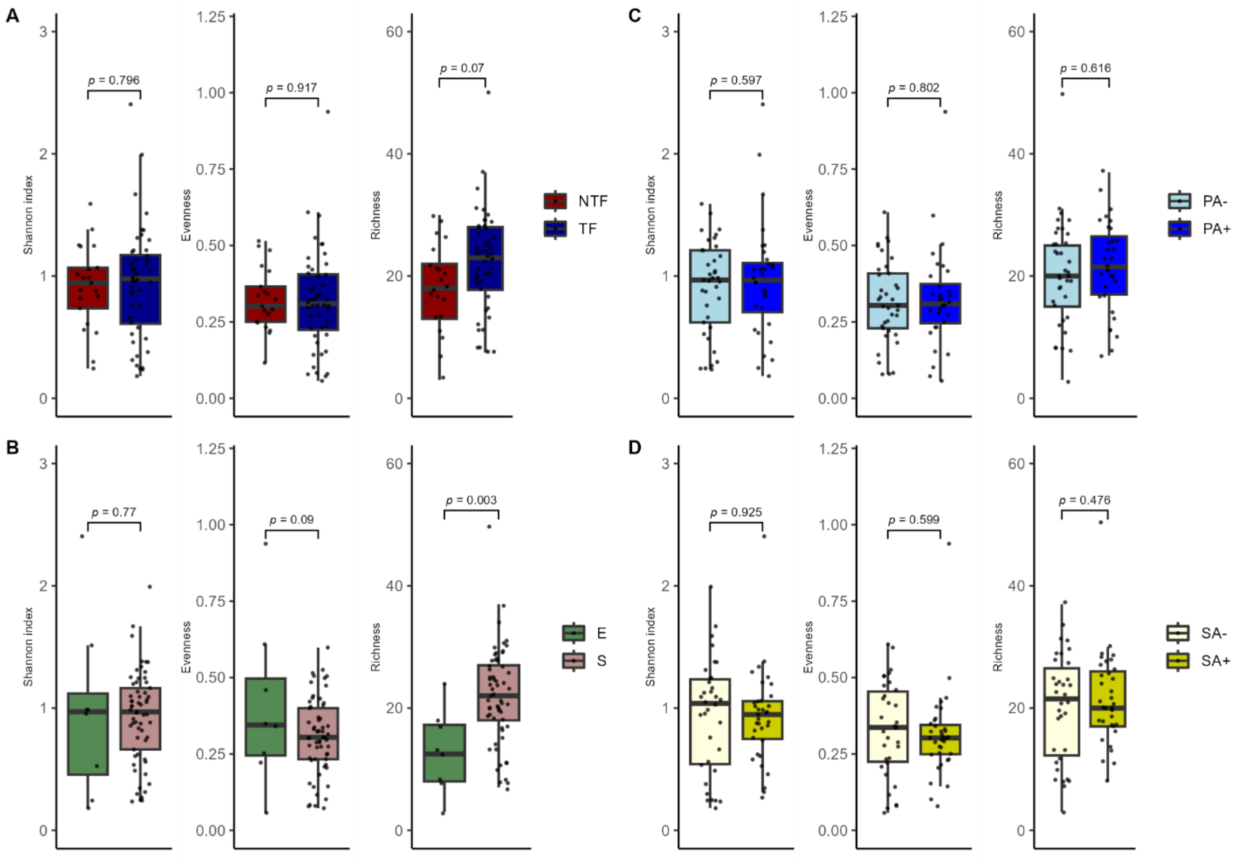

Figure S2. Diversity of the volatilome profile in EBC samples from pwCF under different clinical conditions. The comparison of the Shannon index, evenness, and the richness of the volatiles was made based on the pwCF (n = 72) who were A) on Trikafta (n = 50) or not (n = 22), B) stable (n = 64) vs. those experiencing exacerbation at the time of sample collection (n = 8) C) tested positive (n = 33) or negative (n = 39) for PA D) tested positive (n = 36) or negative (n = 36) for SA. Statistical analysis was conducted using Tukey's HSD test, with a significance level at $p < 0.05$.

Table S1. EBC volume collected during the cough breath sample collection

**Patient ID**	**Approximate volume of EBC collected (µL)**	**Patient ID**	**Approximate volume of EBC collected (µL)**	**Patient ID**	**Approximate volume of EBC collected (µL)**
P01-1	610	P21-1	500	P104	500
P01-2	515	P21-2	500	P126	510
P02-1	500	P22-1	500	P145	500
P02-2	500	P22-2	710	P151	500
P02-3	300	P23	500	P157	660
P03-1	500	P25-1	500	P175	625
P03-2	700	P25-2	500	P198	500
P05	500	P25-3	500	P216	500
P06	500	P26-1	500	P231	500
P08-1	500	P26-2	500	P236	570
P08-2	500	P27	500	P262	500
P09-1	550	P28	500	P269	500
P09-2	800	P29	500	P277	580
P10	505	P30-1	500	P298	580
P11-1	500	P30-2	520	P299	640
P11-2	565	P31-1	500	P363	550
P11-3	500	P31-2	520	P372	500
P12-1	500	P31-3	520	P388	590
P12-2	500	P31-4	500	P397	580
P12-3	500	P32-1	600	P401	500
P13-1	750	P32-2	500	P439	575
P13-2	500	P32-3	500	P462	500
P14-1	500	P33-1	500	P469	600
P14-2	500	P33-2	500	P492-1	500
P14-3	500	P35	500	P492-2	500
P16-1	500	P38	550	P495	500
P16-2	500	P39	500	P496	550
P16-3	500	P41	500	P497	800
P17	500	P46	500	P498	650
P18	500	P61	580	P499	500
P19-1	500	P66	600	P500	500
P19-2	500	P74	500	P515	660
P20	500	P76	505		

EBC; Exhaled Breath Condensate. Volumes collected during cough breath sampling over 5–7 minutes.

Table S2. CF isolates used in the study

Pathogen species	Isolate ID	Patient ID	Culture specimen
PA	P01_PA	P01	Sputum
	P03_PA	P03	Sputum
	P09_PA1	P09	Sputum
	P09_PA2	P09	Sputum
	P12_PA1	P12	CB
	P12_PA2	P12	Sputum
	P14_PA1	P14	Sputum
	P14_PA2	P14	Sputum
	P14_PA3	P14	Sputum
	P31_PA1	P31	Sputum
	P31_PA2	P31	Sputum
	P31_PA3	P31	Sputum
	P35_PA	P35	Sputum
	P200_PA	P200	Sputum
	298_PA	P298	Sputum
	P343_PA	P343	Sputum
	P485_PA	P485	Sputum
	P02_PA	P02	Sputum
	P04_PA	P04	Sputum
	P30_PA	P30	Sputum
	P32_PA	P32	Sputum
SA	P01_SA	P01	Sputum
	P02_SA1	P02	Sputum
	P02_SA2	P02	Sputum
	P02_SA3	P02	Sputum
	P11_SA	P11	Sputum
	P14_SA1	P14	Sputum
	P14_SA2	P14	Sputum
	P14_SA3	P14	Sputum
	P29_SA	P29	Sputum
	P31_SA1	P31	Sputum
	P31_SA2	P31	CB
	P31_SA3	P31	Sputum
	P31_SA4	P31	Sputum
	P31_SA5	P31	Sputum
	P36_SA1	P36	Sputum
	P36_SA2	P36	CB
	P37_SA	P37	Sputum
	P485_SA1	P485	Sputum
	P485_SA2	P485	Sputum
	P309_SA	P309	Sputum
	P33_SA	P33	Sputum
	P37_SA	P37	Sputum
	P12_SA	P12	Sputum
	P30_SA	P30	Sputum
AX	P01_AX	P01	Sputum

	P02_AX1	P02	Sputum
	P02_AX2	P02	Sputum
	P09_AX1	P09	Sputum
	P09_AX2	P09	Sputum
	P37_AX1	P37	Sputum
	P37_AX2	P37	Sputum
	P292_AX	P292	Sputum
	P16_AX	P16	Sputum
	P318_AX	P318	Sputum
Streptococcus spp.	*S. gordonii_1*	P318	Sputum
	S. gordonii_2	P03	CB
	S. anginosus	P03	CB
	S. anginosus	P38	CB
	S. salivarius	P03	CB
	S. salivarius	P35	Sputum
	S. parasanguinis	P11	Sputum
GA	P25_GA	P25	Sputum
SM	P318_SM	P318	Sputum
RM	P318_RM	P318	Sputum

PA; *Pseudomonas aeruginosa,* SA; *Staphylococcus aureus,* AX; *Achromobacter xylosoxidans,* GA; *Granulicatella adiacens,* SM; *Stenotrophomonas maltophilia,* RM; *Rothia mucilaginosa,* CB; cough breath
